# Helminth infection and helminth-derived products: A novel therapeutic option for non-alcoholic fatty liver disease

**DOI:** 10.3389/fimmu.2022.999412

**Published:** 2022-10-03

**Authors:** Xi Liu, Yuyun Jiang, Jixian Ye, Xuefeng Wang

**Affiliations:** ^1^ Department of Central Laboratory, The Affiliated Hospital of Jiangsu University, Zhenjiang, China; ^2^ Department of Nuclear Medicine and Institute of Digestive Diseases, The Affiliated Hospital of Jiangsu University, Zhenjiang, China

**Keywords:** helminth infection, helminth-derived products, NAFLD, glycolipid metabolism, inflammation, gut dysbiosis

## Abstract

Non-alcoholic fatty liver disease (NAFLD) is closely related to obesity, diabetes, and metabolic syndrome (MetS), and it has become the most common chronic liver disease. Helminths have co-evolved with humans, inducing multiple immunomodulatory mechanisms to modulate the host’s immune system. By using their immunomodulatory ability, helminths and their products exhibit protection against various autoimmune and inflammatory diseases, including obesity, diabetes, and MetS, which are closely associated with NAFLD. Here, we review the pathogenesis of NAFLD from abnormal glycolipid metabolism, inflammation, and gut dysbiosis. Correspondingly, helminths and their products can treat or relieve these NAFLD-related diseases, including obesity, diabetes, and MetS, by promoting glycolipid metabolism homeostasis, regulating inflammation, and restoring the balance of gut microbiota. Considering that a large number of clinical trials have been carried out on helminths and their products for the treatment of inflammatory diseases with promising results, the treatment of NAFLD and obesity-related diseases by helminths is also a novel direction and strategy.

## Introduction

With the improvement of living standards in modern society, obesity and related metabolic syndrome have become a global epidemic, especially in developed western countries and wealthy regions in other countries. Non-alcoholic fatty liver disease (NAFLD) has become an important cause of modern chronic liver disease (1, 2). NAFLD has a global prevalence of 25.8% (3, 4), and the population with NAFLD is expected to increase by 21% by 2030 (2). Moreover, NAFLD is being diagnosed in a growing number of obese children and adolescents (5). Despite the increasing prevalence of NAFLD, which imposes a health burden on society and a huge economic burden on the medical industry, no medicines are available yet for the treatment of NAFLD (6, 7). Therefore, new therapeutic strategies for NAFLD should be explored.

Since the 1980s, the incidence of allergic diseases and autoimmune diseases has increased significantly in western countries and most modern countries because of the improvement of people’s living environment and the decrease in the incidence of major infectious and parasitic diseases. Accordingly, experts have coined the term “hygiene hypothesis” (8, 9). A consensus among the general public states that helminths are harmful to humans. However, “hygiene hypothesis” suggests that helminth infections can, in some cases, have beneficial effects on the host and their products may be potential therapeutic modalities (10, 11). Parasite can prevent inflammatory, autoimmune, and metabolic diseases through their excretory/secretory products (ESPs), such as extracellular vesicles, glycans, proteins, and microRNAs (10, 12–18). Parasitic infection can induce the type 2 immune response, effectively control the host inflammatory response, promote wound healing, and regulate tissue repair (19, 20). A recent epidemiological investigation manifested that previous schistosome infection is negatively associated with fatty liver and coronary heart disease (CHD) (21). Schistosoma infection may provide new direction for the prevention and treatment of fatty liver and CHD (21). Therefore, the ability of worm infection and/or its products to intervene with NAFLD needs to be determined. In this review, the pathogenesis of NAFLD is discussed, and the effect of helminth infection and their products on the pathogenesis of NAFLD for improved NAFLD was determined.

## Pathogenesis of NAFLD

NAFLD is a progressive disease that describes a continuum of clinical liver abnormalities, showing changes from non-alcoholic fatty liver disease (NAFL) to non-alcoholic steatohepatitis (NASH). In this process, liver fibrosis and cirrhosis gradually progress. Patients with NASH are at high risk for eventual end-stage liver disease and hepatocellular carcinoma (2, 3, 22). NAFLD is histologically defined as more than 5% of hepatocytes, excluding hepatocyte damage caused by alcohol and other specific factors (23). The pathogenesis of NAFLD is complex, and NAFLD is related to metabolic dysfunction. Considering the heterogeneity of NAFLD, experts have introduced the term “metabolism-related fatty liver disease (MAFLD)”, which is considered to be a more accurate expression of the current understanding of NAFLD, that is, hepatic manifestations in the systemic metabolic disorders (24, 25). NAFLD is a disease caused by multiple factors, including overnutrition, obesity, type 2 diabetes mellitus (T2DM), metabolic syndrome (MetS), and genetics (26). These conditions can cause various injuries, such as lipid accumulation in the liver, insulin resistance (IR), glycolipid metabolism disorder, oxidative stress, release of inflammatory cytokines, and changes in the gut–liver axis, affect the occurrence and development of NAFLD (26, 27). Here, the pathogenesis of NAFLD is discussed from the aspects of glucose and lipid metabolism, inflammation, and gut microbiota.

### NAFLD and glycolipid metabolism

T2DM is substantially associated with NAFLD (28). NAFLD, T2DM, and MetS are frequently coexisting. Generally, MetS is a major risk factor for T2DM. Additionally, among the risk factors that induce NAFLD and NASH, MetS is the strongest (29). MetS is affected by various environmental factors mainly because of high-fat and high-carbohydrate diet, resulting in hyperglycemia, hypertension, dyslipidemia, and a high incidence of fatty liver disease. The main feature of MetS is IR, indicating that insulin’s ability to make use of glucose is declining, particularly in non-hepatic tissues such as adipose and muscular tissues. Hyperinsulinemia, a direct manifestation of IR, is caused by increased blood glucose levels caused by decreased glucose utilization, followed by increased insulin compensatory activity (30, 31). Thus, IR is a pathogenic factor for NAFLD.

Considering that obesity is common characteristic in patients with NAFLD (4), obesity along with overnutrition may contribute to various injuries and thus lead to lipid accumulation in the liver (32). Therefore, the mechanisms of hepatic steatosis development driven by excessive hepatic lipid accumulation need to be understood. The main components of lipids or triglycerides (TG) accumulated in the human liver are primarily derived from circulating free fatty acids (FFAs) in adipose tissue (33, 34). *De novo* lipogenesis (DNL) and dietary fat intake are two important pathways for hepatic lipid accumulation (27, 32, 34). Lipolysis is a process in which fat is hydrolyzed into glycerol and fatty acid (FA) under the action of lipase, thus providing energy for the body (32). The lipase that catalyzes TG is a rate-limiting enzyme and is governed by a large number of hormones. When the lipid content in the body exceeds its processing capacity, the synthesis and utilization of TG are out of balance, leading to the accumulation of TG in the liver, which may be the first inducing factor of NAFLD (35). Liver lipid metabolism homeostasis is regulated by multiple mechanisms, such as hormones, metabolic pathways, and signaling pathways, such as phosphatidylinositol 3-kinase(PI3K)/AKT/PTEN pathway in hepatocytes (36), among which insulin signaling plays a crucial role (37). In general, plasma circulating FFA concentration increases during fasting because of insulin signaling and declines after feeding because of the inhibition of lipolysis. By contrast, in the presence of IR, the decomposition of fat is not restricted, thus increasing the degree of steatosis, thus remarkably increasing the level of circulating FFA, leading to hepatic steatosis (27, 32).

The incidence of liver DNL was substantially increased in patients with NAFLD (38, 39). DNL is the process by which excess carbohydrates are converted into FFAs. In this process, the acetyl-coenzyme A (acetyl-CoA) produced by glycolysis is initially converted to malonyl-coenzyme A (malonyl-CoA) through acetyl-CoA carboxylase (ACC) and ultimately to palmitate, a saturated fatty acid. These FFAs can be converted to TG for storage along with glycerol (39, 40). DNL in patients with NAFLD is regulated by insulin and glucose at the transcriptional level and is negatively correlated with insulin sensitivity (41). Two key transcription factors are involved in the regulation of DNL, including the sterol regulatory element binding protein 1C (SREBP1c), also named as sterol regulatory element binding transcription factor 1 (SREBF1), and the carbohydrate regulatory element binding protein (ChREBP). The activation of SREBP1c is mediated by insulin and liver X receptor (LXR), while ChREBP is activated by carbohydrate metabolites (32, 40–43). Hyperinsulinemia caused by insulin resistance increases the activity of SREBP1c. SREBP1c activation results in the transcription of lipogenic genes such as stearoyl-CoA desaturase 1 (SCD1) and stimulates elevated liver DNL. Hyperglycemia stimulates ChREBP and further induces the transcription of pyruvate kinase, contributing to the conversion of phosphoenolpyruvate and ADP into ATP and pyruvate, and the decarboxylation of pyruvate into CoA, which is then used in DNL, a synthetic pathway (44, 45). Based on mouse studies, the presence of excess lipids enhance liver DNL, creating a vicious cycle (46).

**Figure 1:**
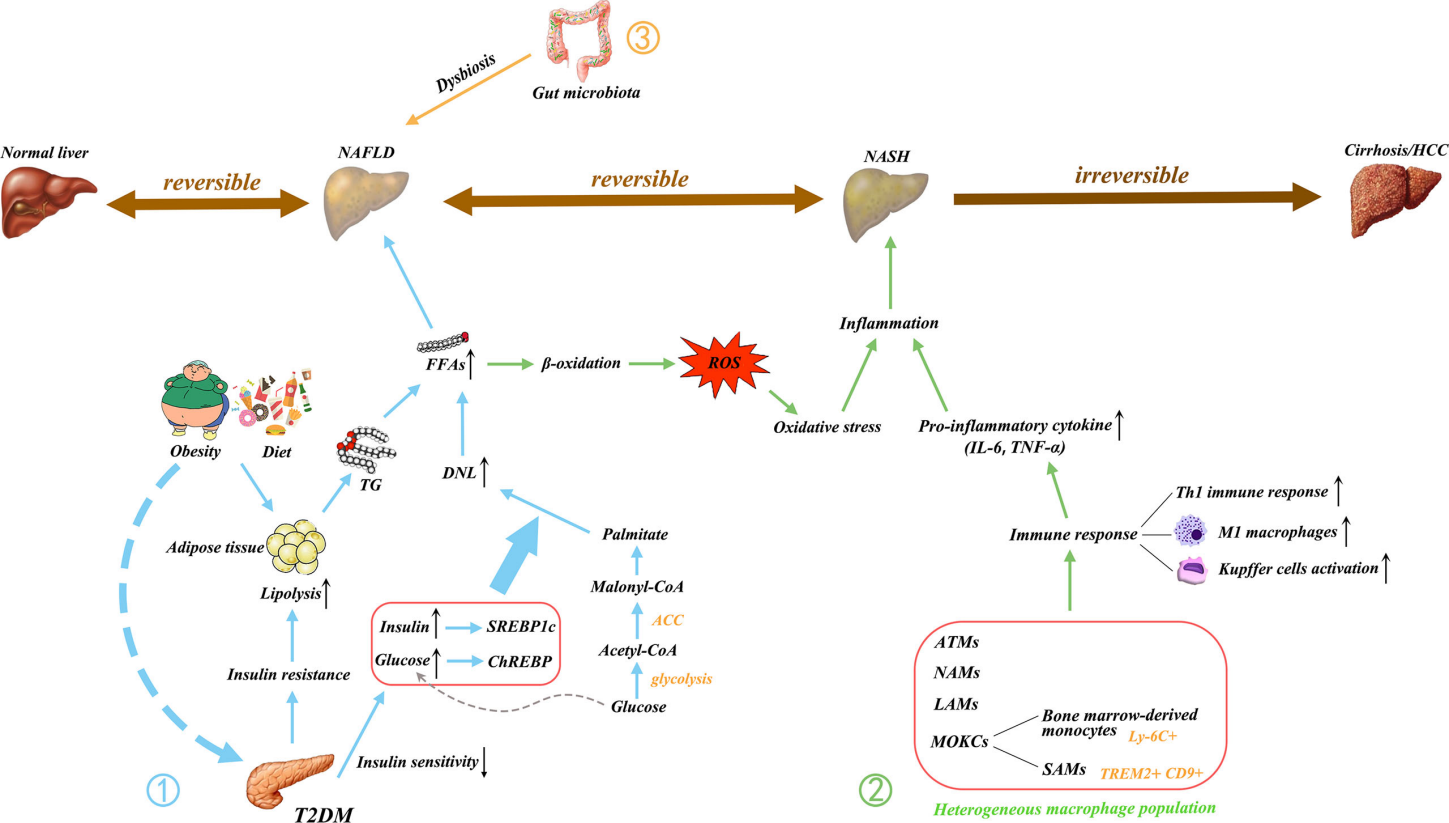
Main pathogenic mechanism of NAFLD. NAFLD is a multifactorial disease; Obesity, diet, and insulin resistance are linked to pathogenesis. ① The increase in free fatty acids (FFAs) is essential to the development of NAFLD. The FFAs produced by the lipolysis of TG in adipose tissue are delivered to the liver, resulting in hepatic steatosis. T2DM causes IR and decreases insulin sensitivity, thus triggering an increase in DNL, which is another major contributor to the increase in FFAs. The excessive delivery of FFAs to the liver upregulates β-oxidation and promotes lipotoxicity, leading to oxidative stress. ② In addition, the activated immune cells secrete pro-inflammatory cytokines. These processes initiate hepatic inflammation and contribute to the transition from NAFLD to NASH. ③ Gut microbiota dysregulation has also been implicated in the pathogenesis of NAFLD. Gut dysbiosis disrupt the intercellular tight junctions, thus allowing the entry of bacterial lipopolysaccharide into the systemic circulation and increasing liver input. Ultimately, it results in liver exposure to inflammatory mediators. NAFLD, nonalcoholic fatty liver disease; NASH, non-alcoholic steatohepatitis; HCC, hepatocellular carcinoma; FFAs, free fatty acids; TG, triglyceride; T2DM, type 2 diabetes mellitus; IR, insulin resistance; DNL, *de novo* lipogenesis; LPS, lipopolysaccharide; ROS, reactive oxygen species; IL, interleukin; TNF, tumor necrosis factor; Th1, T helper type 1; M1, classically activated macrophage; ATMs, adipose tissue macrophages; NAMs, NASH-associated macrophages; LAMs, lipid-associated macrophages; MOKC, monocyte-derived KCs; SAMs, scar-associated macrophage; TREM2, triggering receptors expressed on myeloid cells 2; CD, cluster of differentiation; CoA, coenzyme A; ACC, acetyl-CoA carboxylase; SREBP1c, sterol regulatory element binding protein 1C (SREBP1c); ChREBP, carbohydrate regulatory element binding protein.

A diet in high calories, fat, and sucrose is closely associated with the occurrence of NAFLD and can promote the synthesis of FAs (4). Sucrose can be decomposed into fructose and glucose in the gut. Fructose is essential because of its ability to activate the key transcription factor SREBP1c and ChREBP in liver DNL (47). Although both glucose and fructose are metabolically regulated in the liver, fructose metabolism is more detrimental, because fructose can be directly extracted from the portal vein circulation and then transported to the liver. As a result, hepatocytes are exposed to higher concentrations of fructose than other tissues (47–49). Fructose activates ChREBP by increasing the intracellular concentration of fructose-1-phosphate and enhancing glycolysis flux. ChREBP, together with SREBP1c, induces the increased expression of acetyl-CoA carboxylase (ACC) and fatty acid synthase (FAS), thus increasing the very low-density lipoprotein (VLDL) TG secretion and DNL (35, 50–52). Therefore, abnormal glucose lipid metabolism can lead to NAFLD (**Figure 1**).

### NAFLD and inflammation

In NAFLD, inflammation may be chronic, and liver biopsy may be ignored to some extent, but nearly one-third of patients will develop from simple fatty liver to NASH (35). Excess lipids are ectopically deposited in multiple organs and tissues and impair their function because of overnutrition. In NASH, lipotoxicity caused by excess FFAs delivered to the liver can upregulate mitochondria β oxidation, thus increasing reactive oxygen species (ROS) generation, and inducing oxidative stress. Oxidative stress is a prominent driver of hepatic inflammation (53–55).

The inflammatory response generally occurs after the appearance of tissue damage, and after liver injury in patients with NAFLD, a large number of immune cells (e.g., resident and recruited macrophages, dendritic cells, neutrophils, monocytes, innate lymphoid cells, and mast cells), endothelial cells, and liver parenchymal cells participate in the inflammatory response. Resident liver macrophages such as Kupffer cells (KC), endothelial cells, and liver parenchymal cells have surface receptors that recognize DAMPs and PAMPs, which can bind the receptors and induce the synthesis and release of inflammatory mediators, leading to the recruitment of inflammatory immune cells into the damaged liver tissue (26, 56–59).

The innate immune response triggers hepatic inflammation, thus inducing disease progression toward NASH. Macrophages play a central role. Macrophages that are stimulated by different environmental factors are activated to transform into two distinct functional phenotypes, namely, the classically activated macrophages (M1) and the alternatively activated macrophages (M2) (60). However, this M1/M2 paradigm is only suitable for *in vitro* research (61). Instead of fixed phenotypes *in vivo*, macrophages exhibit high plasticity and can adopt different activation states according to the environment (62, 63). Adipose tissue macrophages (ATMs) accumulate in obese individuals, causing chronic low-grade inflammation. Long-term chronic inflammation leads to insulin resistance and metabolic imbalances. Kratz and colleague found that ATMs from obese humans converts “metabolically activated” phenotype, which is distinct from M1 or M2 markers. These metabolically activated macrophages regulate the balance between cytokine production and lipid metabolism and are driven by independent pro-inflammatory and anti-inflammatory pathways (64). Metabolic disease-specific macrophage phenotypes are also found in NAFLD. Xiong and colleague conducted single-cell RNA sequencing analysis and found that NASH-associated macrophages (NAMs) are highly expressive of triggering receptors expressed on myeloid cells 2 (Trem2) (65). These NAMs are also associated with disease severity and are highly responsive to drug and dietary interventions (65). KCs are replaced by bone marrow-derived macrophages and thus reduced in MAFLD. These recruited macrophages in the liver of MAFLD have two different activation states, one similar to homeostatic KCs and the other to lipid-associated macrophages (LAMs) from obese adipose tissues (66). These LAMs express NASH patient marker osteopontin, which is associated with fibrosis development (66). Compared with the traditional M1/M2 phenotype, the macrophage pool has considerable heterogeneity and requires more specific macrophage-targeting strategies in MAFLD. Tran and colleague also found that liver-resident KCs are replaced by Ly-6C^+^ monocytes during NASH (67). These monocyte-derived KCs (MoKCs) reduce hepatic triglyceride storage, increase inflammation, promote liver damage, and are linked to disease progression during NASH (67). Seidman et al. (68) also identified a subset of MoKCs that resembles scar-associated macrophage (SAM) phenotype. These SAMs express Trem2 and CD9 and are also profibrogenic in liver cirrhosis (69). A dual C-C chemokine receptor type 2 and 5 dual antagonist (named cenicriviroc) can inhibit macrophage infiltration and antifibrosis in NASH animal models. Cenicriviroc entered phase 2b trial in the CENTAUR and improved antifibrosis compared with that in the placebo group (70). Thus, targeting macrophages as inflammation mediators can improve disease progression in NASH or NAFLD. The inflammatory response in the liver promotes the progress in NAFLD (**Figure 1**).

### NAFLD and gut microbiota dysregulation

Another well-recognized risk factor in the progression of NAFLD is the gut microbiota. Extensive research has confirmed that the gut microbiome of patients with NASH and normal subjects have different characteristics. The liver and the gut are inextricably linked to some extent through the “gut-liver axis”, which is an important two-way communication pathway between the gut and the liver. The products of the liver, such as bile acids, can influence the composition of the intestinal flora, the integrity of the intestinal barrier, and intestinal permeability; in turn, the intestinal microbiota influences bile acid synthesis and glycolipid metabolism in the liver (71, 72). Changes in the abundance and diversity of gut microbes are closely related to the progression of NAFLD. Each stage of NAFLD has its special gut microbiota signature (73, 74). Notably, the microbes, bacterial lipopolysaccharides (LPS), and the metabolites of the microorganisms, can affect the function of the liver (75). Thus, the enterohepatic axis plays a non-negligible role in NAFLD and is an effective and important target for the prevention and treatment of NAFLD in the future.

NAFLD is associated with dysbiosis and intestinal bacteria overgrowth (76, 77). Dysbiosis occurs when the beneficial and harmful bacteria in the gut are in imbalance qualitatively and quantitatively, generating a pathological combination. Many researchers speculated that differences in gut bacterial composition cause obese individuals to develop NAFLD. For example, obesity was replicated by transferring the gut microbiota from obese mice or humans to germ-free mice (78). In NAFLD, Firmicutes and Proteobacteria were increased, and Bacteroidetes were reduced at the phylum level. Ruminococcaceae and Rikenellaceae were decreased, and Enterobacteriaceae was increased at the family level. *Anaerosporobacter, Faecalibacterium, Eubacterium, Prevotella*, and *Coprococcus* were reduced, and *Peptoniphilus, Dorea*, and *Escherichia* were increased at the genera level (79). Therefore, fecal microbiome transplantation (FMT) that regulates the gut microbiome can be used to treat NAFLD. Xue and colleague found that FMT can improve intestinal microbiota disorders, thereby reducing fat aggregation and relieving fatty liver. Moreover, FMT improves the reconstruction of gut microbiota in lean NAFLD compared with that in obese patients with NAFLD (80). However, Craven et al. (81) found that allogenic or autologous FMT did not improve IR and hepatic proton density fat fraction but allogenic FMT decreased the small intestinal permeability in patients with NAFLD. Indeed, altered intestinal permeability is associated with liver disease (81). The intestinal mucosal barrier prevents the invasion of bacteria and the absorption of toxins. The mechanical barrier, also known as the physical barrier, is of utmost importance among the intestinal mucosal barriers and rests on the physiological structure of the mucosal epithelium, the lamina propria, and the mucosal base, where the intestinal epithelial cells are tightly arranged by cell junctions. Tight junction protein (TJP), including Zonula Occludens (ZOs), occluding, and claudin, connect the intestinal epithelial cells and maintain the integrity of the intestinal barrier (82). The dysbiosis of the gut microbiota leads to impaired TJP and the disruption of the tight junction between intestinal cells, thus increasing intestinal permeability (83). Under such circumstances, the LPS of gram-negative bacilli is translocated from the intestine to the portal system, and bacterial translocation leads to the exposure of the liver to inflammatory mediators (84, 85). HFD can induce proinflammatory signal, increase intestinal permeability, and cause the development of severe steatohepatitis (86). In comparison with healthy controls, patients with NAFLD increase intestinal permeability. Inflammation and early liver damage alter intestinal permeability in patients with NAFLD (87). Gut dysbiosis caused by inflammasome deficiency leads to the abnormal accumulation of bacterial metabolites in the portal circulation. Liver exposure to high concentrations of portal system products, especially pre-conditioned by lipid accumulation in hepatocytes by excessive nutrient, make it vulnerable to the development of NAFLD/NASH (88). The dysregulation of gut microbiota involved in NAFLD pathogenesis in shown in **Figure 1**.

It is worth noted that white adipose tissue (WAT) is an inflammatory organ where adipocytes interact with immune cells to maintain tissue homeostasis. In general, IL-10, IL-4, and other anti-inflammatory cytokines secreted from T regulatory cells (Tregs) and eosinophils polarize ATMs towards M2 or alternatively activated macrophages, thereby maintaining a tolerogenic phenotype (89, 90). However, excessive nutrition leads to WAT amplification and fat cell hypoxia, followed by chemokine production to induce immune cell infiltration and IL-6, TNF-α, and IL-1β release, resulting in a low-grade inflammatory response in obese individuals (91). Moreover, obesity, T2DM, and other environmental factors can alter intestinal permeability, causing gut-derived endotoxins to penetrate into the circulatory system, affecting liver lipid deposition, and accelerating liver inflammation and fibrosis processes in NAFLD (92).

### Parasite-mediated protection against NAFLD

Helminth infections can antagonize allergic and autoimmune disorders, as suggested by the hygiene hypothesis (8, 93, 94). However, helminth-mediated protection is not limited to autoimmune and allergic diseases. Recent studies have found that chronic helminth infection or helminth-derived products have beneficial effects on host metabolism and improve insulin resistance and T2DM (10). NAFLD is closely related to insulin resistance, T2DM, and MetS. Therefore, helminth may provide the protection against NAFLD.

### Parasite regulates glycolipid metabolism in NAFLD

Considering that NAFLD is closely related to disorders of glycolipid metabolism, the effective intervention of metabolic dysfunction can further influence the development of NAFLD. Numerous mouse experiments and several human studies have confirmed that helminth infection and its derived molecules attenuate obesity, improve IR, glucose tolerance, and MetS (15, 95). *Nippostrongylus brasiliensis* (*N. brasiliensis*) infection decreases weight gain and adipose tissue mass, relieves hepatic steatosis, reduces the expression of key lipogenic enzymes, and improves glucose metabolism in HFD mice (96). *Schistosoma japonicum* (*S. japonicum*) infection upregulates glycolysis-related genes, such as Ldha, Glut4, Pkm2, Glut1, Pfkfb3, Aldoc, HK2, and Pfk, and downregulates gluconeogenesis gene G6pc in mouse liver. Furthermore, *S. japonicum* infection downregulates FA synthesis genes and lipid uptake gene and upregulates FA oxidation-related gene. SEA-stimulated macrophages showed increased gene expression related to glycolysis and FA oxidation but decreased gene expression related to gluconeogenesis, FA synthesis, and lipid uptake. Therefore, *S. japonicum* infection promotes the catabolism of glycolipids and inhibits their anabolic metabolism in mouse livers, possibly *via* the AMPK, AKT, and mTORC1 pathways in macrophages (97). The mice in *S. japonicum* infection reduces miR-802 and lipid metabolism. The reduced miR-802 decreases hepatic lipogenesis by AMPK phosphorylation. Sjp40, a main component of SEA from *S. japonicum* binding with CD36 on hepatocytes to suppress miR-802, leading to the activation of AMPK pathway and mitigation of lipogenesis in liver. Sjp40 shows therapeutic potential in treating obesity-related fatty liver (14). da Silva Filomeno CE et al. demonstrated that *Schistosoma mansoni* (*S. mansoni*) infection improves glucose tolerance, body mass and liver steatosis in HFD mice but aggravates pathological damage to the liver in mice (98). Similarly, *Taenia pisiformis* infection decreases body weight and cholesterol level in obese rabbits and changes the metabolic features in rabbits (99). Bhargava et al. further demonstrated that lacto-N-fucopentaose III (LNFPIII) or soluble egg antigen (SEA) from *S. mansoni* improves glucose tolerance and insulin sensitivity, suppresses lipogenesis in the liver, and provides a strong protective effect against HFD-induced hepatosteatosis in mice. The upregulation of IL-10 in activated macrophages and dendritic cells and extracellular signal-regulated kinase (Erk)-Ap1 pathway in liver mediate the effects of LNFPIII on glucose and lipid metabolic pathway, respectively (15). The synthetic small molecule analogues (SMAs) of ES-62 from *Acanthocheilonema viteae *(*A.viteae*) exhibit antimetabolic disorder activity in mice on a high calorie diet (HCD) by reducing fasting glucose levels in male mice and mitigating ileal villus length and liver fibrosis caused by HCD in female mice. Thus, SMAs have the potential to prevent or treat metabolic disorders associated with obesity (100).

**Figure 2:**
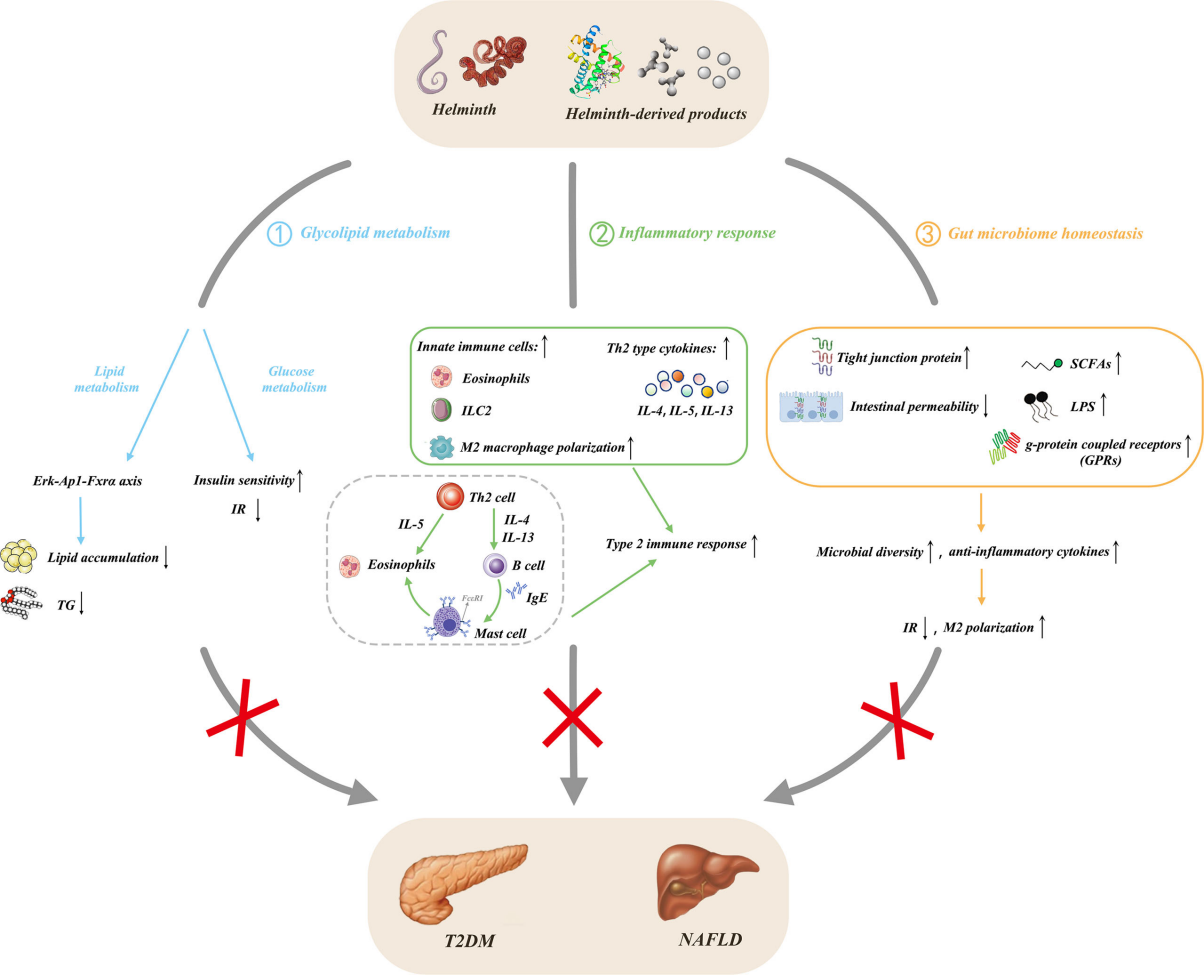
Protective effects of helminths and their products in NAFLD. ① Helminth infection and its derived products regulate glucose metabolism, alleviate insulin resistance, and improve insulin sensitivity; they also affect lipid metabolism and reduce lipogenesis through the Erk-Ap1-Fxrα axis; ②secrete anti-inflammatory cytokines by inducing type 2 immunity and M2 macrophage polarization; ③ influence the composition of gut microbiota through the liver-gut axis, increase the level of fecal SCFAs and upregulate the expression of its main receptors, GPRs, thereby coordinating multiple signaling pathways to prevent obesity. Meanwhile, the increase of tight junction proteins leads to the decrease of intestinal permeability and the expansion of the dominant bacteria, causing the increase of intestinal microbial abundance and diversity. NAFLD, nonalcoholic fatty liver disease; T2DM, type 2 diabetes mellitus; TG, triglyceride; IR, insulin resistance; IL, interleukin; LPS, lipopolysaccharide; Erk, extracellular signal-regulated kinase; AP-1, activator protein-1; Fxrα, farnesoid X receptor alpha; Th2, T helper type 2; M2, alternatively activated macrophage; ILC2, type 2 innate lymphoid cell; Ig, immunoglobulin; SCFAs, short chain fatty acids; GRPs, g-protein coupled receptors.

Human studies also found that helminth infection improves glycolipid metabolism. Rennie C et al. found that helminth infection induces lower fasting blood glucose, HbA1c levels, prevalence of MetS, and T2DM than without infection people by using meta-analysis (101). Helminth infections generally improve metabolic function, but different species have different effects, and *S. mansoni* infection remarkably improves metabolic outcomes (101). Zinsou JF et al. found that individuals infected by *Schistosoma haematobium* (*S. haematobium*) exhibit decreased serum triglyceride (TG), total cholesterol (TC), and high-density lipoprotein (HDL)-C, especially in overweight/obese individuals. TC, HDL-C, LDL-C, and TG levels in serum are inversely correlated with the intensity of *S. haematobium* infection in overweight/obese individuals. *S. haematobium* infection can improve lipid profile in overweight/obese individuals and contribute to the decreased risk of cardiometabolic diseases caused by hyperlipidemia (102). Thus, helminth infection or helminth-derived molecules can improve NAFLD by regulating glycolipid metabolism (**Figure 2**).

### Parasite regulates inflammatory response in NAFLD

Obesity is a chronic low-grade inflammation, also named meta-inflammation, which easily alters glycolipid metabolism and insulin resistance and ultimately results in the development of T2DM (103). Various innate and adaptive immune cells are activated in metabolic organs, especially adipose tissue and liver, which produce pro-inflammatory cytokines, such as IL-6 and TNF-α, resulting in insulin resistance and metabolic dysregulation (104). Helminth infection potently induces type 2 immune responses characterized by Th2 phenotype polarization, secretion of IL-4, IL-5, and IL-13, and type 2 innate lymphoid cells (ILC2s). Helminth infection also induces immune tolerance, which is characterized by the abundance of IL-10 and TGF-β secreted by regulatory T cells (Tregs), regulatory B cells (Bregs), tolerogenic dendritic cells (DCs), and M2 macrophages (105–107). Thus, the tolerable response to helminth infection can inhibit the inflammation of allergic and autoimmune diseases (10) and the inflammation of obesity, T2DM, MetS, and NAFLD (108).

Helminth infection and their products can decrease inflammation in adipose tissue and improve glucose tolerance and weight gain in obese individuals and mice (10). *Litomosoides sigmodontis* (*L. sigmodontis*) infection resulted in the recruitment of eosinophil and M2 macrophages in epididymal adipose tissue (EAT) and promoted glucose homeostasis in HFD mice. *L. sigmodontis* antigen (LsAg) treatment also improved glucose tolerance, reduced inflammatory responses, and promoted insulin signaling in EAT. However, LsAg-mediated glucose homeostasis was independent of Foxp3 Tregs (109). *Heligmosomoides polygyrus* (*H. polygyrus*) infection improved insulin sensitivity and reduced fat accumulation in the liver and obesity-related inflammation in HFD mice. Treg frequency and suppressor function in adipose tissue increased after *H. polygyrus* infection. *H. polygyrus* contributed to improve weight gain and MetS by regulating adipose tissue Tregs in obese mice (110). *N. brasiliensis* infection increased IL-5 and IL-13 levels in visceral adipose tissue (VAT) and promoted insulin sensitivity in HFD mice. IL-33 promoted ILC2s, produced IL-5 and IL-13, promoted eosinophils, and alternatively activated macrophages in VAT, thus promoting metabolic homeostasis (111). Similarly, *S. mansoni* infection increased eosinophils and M2 macrophages in white adipose tissue (WAT), enhanced insulin sensitivity, and decreased weight gain, fat mass, and adipocyte size. SEA injection also induced type 2 immune responses in WAT and liver and promoted whole-body metabolic homeostasis (112). Individuals infected with *Strongyloides stercoralis* infection (Ss+) showed a decrease in various pro-inflammatory cytokines and chemokine levels in plasma, but anthelmintic treatment restored the reduced inflammatogenic cytokines and chemokines (113). Peroxisome proliferator-activated receptor-γ (PPAR-γ), a key master transcription factor for adipogenesis, stimulates lipogenic enzyme expression, thereby promoting lipid storage in adipose tissue (114). PPAR-γ suppress inflammation, resulting in lower TG/HDL cholesterol ratio when activated by its agonist pioglitazone in diabetic patients (115, 116). Lysophosphatidylcholine (LPC) from *S. mansoni* induces M2 macrophage polarization by increasing PPARγ expression in macrophages (117). The T2 ribonuclease omega-1 (ω1) is an RNase that degrades host RNA. ω1 from *S. mansoni* treatment improves glycolipid metabolism, and this phenomenon is associated with the induction of type 2 immunity in HFD mice. Th2 cells, eosinophils, and M2 macrophages are increased in the WAT of mice after ω1 injection. Furthermore, ω1 induced type 2 immunity, thus promoting whole-body metabolic homeostasis by suppressing food intake *via* signal transducer and activator of transcription 6 (STAT6)-independent mechanism in obese mice (118). Surendar and colleagues demonstrated that HFD increased the numbers of CD4^+^ and CD8^+^ T cells and production of IFN-γ and IL-17 in the adipose tissue. Adiponectin can inhibit the differentiation of Th1 and Th17 cells and decrease the level of IFN-γ and IL-17 in HFD mice. LsAg from *L. sigmodontis* treatment increased adiponectin level and decreased Th1 and Th17 cell frequencies in the adipose tissue of mice, thereby improving obesity and insulin resistance in obese mice (119). Similarly, LNFPIII from *S. mansoni* improved glucose homeostasis, and this process is partially mediated by IL-10 secreted from DCs and macrophages (15). Notably, SJMHE1, an HSP60-derived peptide from *S. japonicum*, could induce the CD4^+^CD25^+^ Treg amplification *in vivo* and *in vitro* (120). Furthermore, SJMHE1 suppresses delayed-type hypersensitivity and collagen-induced arthritis, asthma, and colitis by regulating the balance of Th cells (16–18, 121, 122); it also promotes peripheral nerve repair through inducing M2 macrophages (123). Considering that that NAFLD progression is caused by lipid accumulation in liver to steatohepatitis characterized by inflammation, whether SJMHE1 could be potential to intervene in NAFLD needs to be evaluated. Helminths and their products would be promising to contribute to innovative therapy for obesity-related fatty liver by regulating inflammation (**Figure 2**).

### Parasite regulates dysbiosis of gut microbiota in NAFLD

Obesity, diabetes, and NAFLD have dysregulation of the gut microbiota (124, 125). Reducing the diversity and abundance of gut microbiota is often associated with obesity, IR, MetS, and NAFLD (79, 126, 127). Moreover, helminth infection can change the composition and function of gut microbiota, improve host metabolism, reduce systemic inflammation, and promote insulin sensitivity (128, 129). Khudhair and colleagues found that *N. brasiliensis* infection remarkably alters the composition in gut microbiota at both the phylum and order level, induces type 2 immune responses in adipose tissue, liver, and gut, and promotes glucose homeostasis, thereby preventing T2D (129). *N. brasiliensis* infection decreases the abundance of Verrucomicrobia and TM7 phyla and increases the richness of Proteobacteria at the phylum level in mice fed with high glycemic index (HGI) diet. *N. brasiliensis* infection increases the abundance of Clostridiales and Desufovibrionales at the order level in high-fat (HF) mice. Furthermore, *N. brasiliensis* infection elevates short chain fatty acid (SCFA) levels, which can be beneficial for regulating inflammation and promoting insulin sensitivity (129). SCFAs can maintain gut integrity, immune, and metabolism homeostasis, and it can regulate appetite, weight gain, and glycolipid metabolism (130, 131). Pace et al. found that *Strongyloides venezuelensis* infection modified gut microbiota, most notably by increasing Lactobacillus spp in HDF mice. This alteration of microbiota increased anti-inflammatory cytokine production, induced M2 macrophage polarization in adipose tissue, increased tight junction protein expression in intestinal cells, and reduced LPS level in serum, thereby promoting glucose metabolism homeostasis (128). ESPs from *N. brasiliensis* treatment improve glucose tolerance, and decrease weight gain, and induce type 2 immune response in HGI mice. *N. brasiliensis* ESPs also alter the composition of gut microbiota in mice fed with HGI diet at the phylum and order levels. *N. brasiliensis* ESP treatment increases the abundance of Bacteroidetes and Patescibacteria phylum, reduces the abundance of Actinobacteria phylum, which is related to the HGI group. ESPs from *N. brasiliensis* adult worm treatment also increase the abundance of Lactobacillales and Saccharimonadales but decrease Coriobacteriales abundance. *N. brasiliensis* ESPs induces immune response and gut microbiota changes, which confer protection against abnormal glucose metabolism in mice (132). Similarly, ESPs from the larval *Echinococcus granulosus *(*E. granulosus*) treatment can mitigate damage to the intestinal barrier caused by high-fat (HF) diet, increase the expression of zonula occludens-1 (ZO-1), relieve the translocation of bacterial endotoxins and gut inflammation, and attenuate HF diet-induced microbiota dysbiosis, thereby antagonizing obesity-related neurodegenerative diseases (133). ES-62 from *A. viteae* treatment improved a range of inflammatory, metabolic, and microbiome parameters of aging in HCD-accelerated aging mice (134). A phase 1b clinical trial with *Necator americanus* (*N.americanus*) inoculated obesity and MetS individuals is ongoing. The evaluation of the safety and tolerability of infection with *N. americanus*, metabolic and immunological parameters, and the composition of fecal microbiome in this phase 1b clinical trial will provide valuable information about the use of *N. americanus* for the treatment of metabolic diseases (135). Helminths can affect host immune function by secreting various molecules or indirectly by altering the gut microbiota (136). The interactions between helminth and microbota can shape the homeostasis of the immune system, thereby improving metabolic homeostasis and immune balance in obesity, MetS, and NAFLD (**Figure 2**).

## Challenges and prospect

Although helminths and their molecules have beneficial outcomes in obesity, MetS, T2DM, and NAFLD, many challenges remain to be considered. First, helminth infection can cause a series of pathological effects. For example, *S. mansoni* infection can cause anemia, malnutrition, growth stunting, progressive liver fibrosis, and portal hypertension (137). Furthermore, liver flukes *Opisthorchis viverrini* (*O. viverrini*) and *Clonorchis sinensis* infection can induce cholangiocarcinoma (138, 139), and *S. haematobium* infection can cause squamous cell carcinoma of the bladder (140). In addition to the above harmful effects, parasitic infections can also weaken vaccination effects and increase susceptibility to other pathogens (141, 142). Although *O. viverrini* infection improved insulin resistance and liver lipid accumulation in high-fructose diet hamsters, the animals showed severe NAFLD as indicated by histopathological analysis (143). Helminths or their products that induce type 2 immunity may increase the incidence of asthma. Caraballo et al. reported that *Ascaris lumbricoides* tropomyosin has strong allergenic activity (144). Furthermore, the incidence of asthma increases in obese patients (145). Therefore, this issue should be considered in helminth molecules for the treatment of obesity-related diseases, including NAFLD. In addition, the pharmacokinetics of helminth molecules and the mechanism in which they reach target tissues are also challenges. Helminth proteins are only present for a few hours (10). Similar to most active molecules, nanocarriers for the delivery of helminth molecules may increase their half-life and improve their activity *in vivo* compared with free molecules (146).

Recently, extracellular vesicles (EVs) released by helminths at various life stages can package and deliver immune modulators to host target cells, thereby manipulating the host immune response and exerting immunomodulatory effects (147). Thus, helminth EVs have anti-inflammatory therapeutic potential. *Fasciola hepatica* EVs prevent DSS-induced ulcerative colitis (148), and *Trichinella spiralis* EVs decrease the severity of DSS- and TNBS-induced colitis (149, 150). Whether the immunomodulatory propensity of helminth-derived EVs can be used for the treatment of obesity, T2DM, MetS, and NAFLD must be explored in the future. Current research mainly focuses on animal experiments, but the immunology and gene expression patterns in inflammatory diseases greatly differ between human patients and animal models (151, 152). Thus, future research must focus on helminth molecules to intervene in NAFLD-related diseases in human clinical trials.

Despite these challenges, helminth molecules for evolutionary stress selection are effective and safe, especially for NAFLD treatments without effective drugs. Many nature-inspired drugs, such as venom from insects and reptiles, have been used for the treatment of various diseases for millennia (153). Helminths have been included in this list as other nature-inspired drugs and warrant further studies. Most reports on helminth regulation of NAFLD-related diseases, including obesity, T2DM, and MetS, focused on nematodes and trematodes, such as *N. brasiliensis*, *H. polygyrus*, *L. sigmodontis*, *S. venezuelensis*, *S. mansoni*, and *S. japonicum* (95). In the case of cestodes, only *T. pisiformis* interventions were seen in obese rabbits (99). In addition, ESPs from larval *E. granulosus* can reverse HF-induced gut barrier dysfunction and microbiota dysbiosis, thereby protecting against obesity-associated neurodegenerative diseases (133). Helminths and their molecules regulated NAFLD-related diseases as shown in **Table 1**. Only one study reported that ESPs from adult worms and infective third-stage larvae of *N. brasiliensis* can improve glucose tolerance and attenuate body weight gain in HGI mice (132). Nevertheless, helminth intervention for NAFLD-related diseases is still in its infancy, and whether helminths can treat metabolic disorders in obesity, T2DM, MetS, and NAFLD remains unclear. However, studies on controlled human infection (CHI) with *N. americanus* and single-sex *S. mansoni* are ongoing (154–156). Clinical trials using CHI or helminth molecules to treat inflammatory metabolic diseases, such as obesity, T2DM, NASH and NAFLD, will provide new insights and key messages in the future. Clinical trials of the porcine whipworm Trichuris suis ova (TSO) in the treatment of Crohn’s disease demonstrated that patients received a single dose of 7500 TSO still showed tolerance and did not display any short- or long-term treatment-related side effects (157). Clinical trials of experimental helminths in the treatment of inflammatory diseases have been comprehensively reviewed and have yielded promising results (10). Recently, a phase 1b clinical trial by using the larvae III stage of *N. americanus* in the treatment of obesity and MetS is underway to evaluate the safety and tolerability of hookworm (135). These clinical trials lay the foundation for the development of next generation of therapies against obesity-related diseases, including NAFLD.

**Table 1:** Helminths or helminth-derived products suppress obesity, T2DM, and NAFLD-related diseases.

Class	Helminth species	Helminth or Helminth-derived products	Models	Regulatory effect	Reference
Trematoda	*Schistosoma japonicum*	infection	C57BL/6 mice	glycolysis-related gene**↑**, FA oxidation-related gene**↑**, gluconeogenesis gene**↓**, FA synthesis genes**↓**, lipid uptake gene**↓**	(95)
		SEA-stimulated macrophages	RAW264.7 cells	glycolysis and FA oxidation genes**↑**, gluconeogenesis gene**↓**, FA synthesis gene**↓**, and lipid uptake gene**↓**	(95)
		Sjp40	HFD C57BL/6 mice	lipid metabolism**↓**, lipogenesis in liver**↓**	(14)
	*Schistosoma mansoni*	infection	HFC C57BL/6 mice HFD C57BL/6 mice	glucose tolerance**↑**, body mass**↓**, liver steatosis**↓**, body weight gain**↓**, fat mass gain**↓**, adipocyte size**↓**, insulin sensitivity**↑**	(96, 110)
		LNFPIII	HFD C57BL/6 mice	glucose tolerance**↑**, insulin sensitivity**↑**, lipogenesis in the liver**↓**, liver steatosis**↓**	(15)
		SEA	HFD C57BL/6 mice	glucose tolerance**↑**, insulin sensitivity**↑**, lipogenesis in the liver**↓**, liver steatosis**↓**	(110)
		LPC	macrophages from C57BL/6 mice	PPARγ expression**↑**, lipogenic enzyme expression**↑**, lipid storage in adipose tissue**↑**, M2 macrophage polarization**↑**	(115)
		ω1	C57BL/6J mice	Th2 cells, eosinophils, and M2 macrophages in WAT**↑**, type 2 immunity**↑**, food intake**↓**, whole-body metabolic homeostasis**↑**, inflammation**↓**	(116)
	*Schistosoma haematobium*	infection	overweight/obese individuals	serum TC, HDL-C, LDL-C, and TG levels**↓**, risk of cardiometabolic diseases caused by hyperlipidemia**↓**	(100)
Cestoda	*Taenia pisiformis*	infection	HFD New Zealand rabbits	body weight**↓**, cholesterol level**↓**, liver and testicular weight**↑**, submandibular gland weight**↓**, body fat**↑**	(97)
	*Echinococcus granulosus*	ESPs	HFD C57BL/6J mice	intestinal barrier damage**↓**, ZO-1 expression**↑**, bacterial endotoxins translocation**↓**, gut inflammation**↓**, HFD-induced microbiota dysbiosis**↓**	(131)
Nematoda	*Nippostrongylus brasiliensis*	infection	HFD C57BL/6 or RIP2-Opa1KO mice HGI or HFD C57BL/6 mice	weight gain**↓**, adipose tissue mass**↓**, hepatic steatosis**↓**, glucose metabolism**↑**, fasting blood glucose**↓**, oral glucose tolerance**↓**, eosinophil and Th2 immune response**↑**, altered alpha diversity and microbial richness	(94, 109, 127)
		ESPs	HGI C57BL/6 mice	glucose tolerance**↑**, body weight gain**↓**, type 2 immune response**↑**, affected microbial composition	(130)
	*Acanthocheilonema viteae*	SMAs of ES-62 or ES-62	HCD C57BL/6J mice	fasting glucose levels**↓**, ileum villus length**↑**, liver fibrosis**↓**, type 2 immune response**↑**, visceral adipose tissue dysfunction and gonadal adipocyte hypertrophy in male mice**↓**, gut health**↑**, normalized the gut microbiota	(98, 132)
	*Litomosoides sigmodontis*	infection	HFD BALB/c mice	eosinophil and M2 macrophages in EAT**↑**, glucose homeostasis**↑**	(107)
		LsAg	HFD BALB/c mice HFD C57BL/6J mice	glucose tolerance**↑**, inflammatory responses**↓**, insulin signaling in EAT**↑**, adiponectin level**↑**, Th1 and Th17 cells in the adipose tissue**↓**, insulin resistance**↓,** improves obesity	(107, 117)
	*Heligmosomoides polygyrus*	infection	HFD C57BL/6 mice	weight gain**↓**, insulin sensitivity**↑**, fat accumulation in the liver**↓**, obesity-related inflammation**↓**, Treg frequency and suppressor function in adipose tissue**↑**	(108)
	*Strongyloides stercoralis*	infection	individuals	pro-inflammatory cytokines**↓**, chemokine levels**↓**	(111)
	*Strongyloides venezuelensis*	infection	C57BL/6 mice	Lactobacillus spp**↑**, anti-inflammatory cytokine production**↑**, M2 macrophage polarization in adipose tissue**↑**, tight junction protein expression in intestinal cells**↑**, LPS level in serum**↓**, glucose metabolism homeostasis**↑**	(126)

HFD, high-fat diet; HFC, high-fat chow; FA, fatty acid; RIP2-Opa1KO, pancreatic β cell Opa1 deficiency; HGI, High Glycemic Index diet; ESPs, excretory/secretory products; HCD, high calorie diet; SMAs, small molecule analogues; WAT, white adipose tissue; TC, total cholesterol; HDL-C, high-density lipoprotein cholesterol; LDL-C, low-density lipoprotein cholesterol; TG, triglycerides; ZO-1, zonula occludens-1; ETA, epididymal adipose tissue ↑(increase); ↓(decrease).

## Author contributions

XL, YJ, JY, and XW wrote the manuscript. XL researched the literatures and drew Figures. XW revised the manuscript. All authors contributed to the article and approved the submitted version.

## Funding

This work was supported by a grant from the National Natural Science Foundation of China (81871243), the key research and development programs of Jiangsu Province (BE2017697), the Six Talent Peaks of Jiangsu Province (WSN-009), and Zhenjiang Clinical Research Center of Gynecological Diseases of Traditional Chinese Medicine (SS202204-KFB02).

## Conflict of interest

The authors declare that the research was conducted in the absence of any commercial or financial relationships that could be construed as a potential conflict of interest.

## Publisher’s note

All claims expressed in this article are solely those of the authors and do not necessarily represent those of their affiliated organizations, or those of the publisher, the editors and the reviewers. Any product that may be evaluated in this article, or claim that may be made by its manufacturer, is not guaranteed or endorsed by the publisher.
